# Social Justice and Public Cooperation Intention: Mediating Role of Political Trust and Moderating Effect of Outcome Dependence

**DOI:** 10.3389/fpsyg.2018.01381

**Published:** 2018-08-14

**Authors:** Shuwei Zhang, Jie Zhou

**Affiliations:** ^1^Center for Chinese Public Administration Research, School of Government, Sun Yat-sen University, Guangzhou, China; ^2^Key Laboratory of Behavioral Science, Institute of Psychology, Chinese Academy of Sciences, Beijing, China

**Keywords:** distributive justice, procedural justice, competence-based trust, motive-based trust, outcome dependence

## Abstract

Cooperation is vital to human evolution and the development of society. In addition, social justice is one of humanity’s long pursuits. Based on social exchange theory and system justification theory, we built and tested a comprehensive mediated moderation model of the relationship between social justice and public cooperation intention via the mediation of political trust and with the moderation of outcome dependence. This research consisted of two studies using laboratory experiment (*N* = 320) and field survey (*N* = 1240) methods. Data were collected from participants located in China. The results showed that (1) both competence-based trust and motive-based trust mediated the relationship between social justice (i.e., distributive justice and procedural justice) and public cooperation intention; (2) outcome dependence moderated the relationship between social justice and public cooperation intention; and (3) the moderation of outcome dependence functioned through the mediating effect of competence-based and motive-based trust. The theoretical and practical significance of these findings is discussed.

## Introduction

As is well known, the 125th anniversary edition of *Science* proposed 125 questions facing science, which included the question “How did cooperative behavior evolve?" Moreover, this question about cooperation was listed in the 25 foremost and highlighted questions (Kennedy and Norman, 2005). Public cooperation, through which people cooperate with authorities (i.e., government and/or legal authority) and social institutions (i.e., non-governmental organizations or non-profit organizations), in order to promote the goals of the group and collective well-being, is the most prevalent form of cooperative behavior in modern society (VanVugt et al., 2000; Sullivan et al., 2008). Especially, the voluntary public cooperation is valuable in vertical relationships between people and government, as voluntary cooperation from people endorses the legitimacy of government or authorities (Tyler, 2010; Feldman, 2015). Therefore, one way to break down *Science*’s question about cooperation is “How do people voluntarily cooperate with authorities?" Previous research showed that social justice has a great impact on public cooperation (Rankin and Tyler, 2009; Tyler, 2012, for a review), but the mechanism and the boundary of this effect are still elusive.

### Social Justice and Public Cooperation: Stimulus and Response

Social justice, defined as the perceived adherence to rules or norms that reflect appropriateness in governmental decision contexts (Colquitt and Rodell, 2015; Colquitt and Zipay, 2015), is mainly divided into distributive justice and procedural justice (Sweeney and McFarlin, 1993; Tyler, 2000; Bobocel and Gosse, 2015).

Distributive justice which refers to individual perceived justice in the distribution of resources or outcomes (Törnblom and Kazemi, 2015; Jasso et al., 2016) can arouse public cooperation. For example, distributive justice positively affected both tax compliance attitudes and intentions to comply among small business owners in the Netherlands (Verboon and Goslinga, 2009). On the contrary, Rothmund et al. (2016) found that distributive injustice was associated primarily with participation in political protests, similar to an analysis conducted in Chile (Castillo et al., 2015).

In addition, procedural justice referring to individual perceived justice in the allocation process (Bobocel and Gosse, 2015; Vermunt and Steensma, 2016) are more likely to promote public cooperation. For instance, procedural justice was found to be the key antecedent to public support for policing and cooperation with the police (Sunshine and Tyler, 2003; van Damme et al., 2015; Tyler, 2017). Moreover, procedural justice was strongly associated with legal compliance and public cooperation with law enforcement (Huq et al., 2011; Murphy, 2014; Nagin and Telep, 2017), especially voluntary deference (Tyler et al., 2014). When the outcome was unfavorable, procedural justice completely determined people’s reactions to public policymaking (Wu and Wang, 2013).

Taken together, both distributive justice and procedural justice facilitate effective cooperation and thereby enable superior levels and forms of social coordination. From the psychological perspective, social justice is viewed as a “stimulus,” while public cooperation is viewed as a “response.” What process can we find from stimulus to response?

### Political Trust: Pivotal Mediator of the Social Justice–Public Cooperation Relationship

Political trust, which may be a potential bridge for linking social justice to public cooperation, refers to citizen judgments and evaluations of the trustworthiness of political officeholders, political organizations, and governments (Levi and Stoker, 2000). Our research focuses on the trust in government,^1^ described as the perceived trustworthiness of the government and its sectors (e.g., police force). Perceived trustworthiness of the government has two distinctive components (Li, 2004, 2008; Lu, 2014). One component is competence-based trust, referring to positive expectations of governmental competence. This is the extent to which a citizen perceives a government to be capable, effective, and professional. Another component is motive-based trust, defined as positive expectations of governmental motive. That is the extent to which a citizen perceives a government to be concerned with the well-being of others and to be motivated to act in the public interest (Tyler, 2011b).

Regarding the relationship between political trust and public cooperation, past research showed that political trust was a perfect lubricant for administration by expanding citizens’ willingness to accept government authority (Kim, 2005). A trustworthy government is likely to encourage citizens’ compliance to the extent that the citizens deem legitimate the authority that made the decision (Levi, 1998). If political trust is high, citizens can sacrifice their interests to support the government (Rudolph and Evans, 2005), by means, such as taxpaying, voting, and rule adherence (Scholz and Lubell, 1998; Hetherington, 1999; Tyler, 2011a). Conversely, declining trust in government reduces public support for government action to address a range of domestic policy concerns (Chanley et al., 2000; Hetherington, 2005). Additionally, low levels of political trust are associated with less law compliance within a society (Marien and Hooghe, 2011).

Knowing that both social justice and political trust are positively related to public cooperation, what is the relationship between them? According to fairness heuristic theory, organizational justice has a causal effect on employees’ trust in authority (Lind, 2001; Proudfoot and Lind, 2015). A good example of this was provided by two meta-analytic reviews, which demonstrated that both distributive and procedural justice were positively correlated with trust in one’s organization and supervisor (Colquitt et al., 2001, 2013). More importantly, social exchange theory (SET) research showed that organizational trust was a mediator of the relationship between organizational justice and work outcomes, such as organizational citizenship behavior (Konovsky and Pugh, 1994; Aryee et al., 2002; Cropanzano and Mitchell, 2005; Colquitt et al., 2012, 2013; van Dijke et al., 2018). Although the relationship between justice and trust and the mediating role of trust in the association of justice with certain positive outcomes have only been demonstrated in an organizational context, based on the effects of social justice and political trust on public cooperation, we expect that political trust, following social justice, can boost public cooperation in a social context. Therefore, we propose the following hypotheses:

H1: Competence-based trust mediates the relationship between social justice and public cooperation intention, such that:
H1a: Competence-based trust mediates the relationship between distributive justice and public cooperation intention;
H1b: Competence-based trust mediates the relationship between procedural justice and public cooperation intention.
H2: Motive-based trust mediates the relationship between social justice and public cooperation intention, such that:
H2a: Motive-based trust mediates the relationship between distributive justice and public cooperation intention;
H2b: Motive-based trust mediates the relationship between procedural justice and public cooperation intention.

We measured public cooperation intention instead of public cooperation behavior in our research because when actual behavior is difficult to obtain, the behavioral intention is its closest predictor (Ajzen and Fishbein, 1980; Zhou and Wang, 2012; Zhang, 2017).

### Outcome Dependence: Core Moderator of the Social Justice–Public Cooperation Relationship

So far, we have focused on social justice and its correlations with political trust and public cooperation. However, justice and injustice are two sides of the same coin; low levels of social justice means social injustice (Colquitt et al., 2015; Cropanzano et al., 2017). In the theoretical perspective, when people experience social injustice, they will reduce their cooperation with the government according to the aforementioned analysis and hypotheses, but in practice, perceived injustice does not inevitably trigger political protest (e.g., collective action) (e.g., Zhang et al., 2010). In most cases, suffering people and members of disadvantaged groups have displayed “the tolerance of injustice,” (Martin, 1986; Wright, 2001) so which factors can facilitate or alleviate the relationship between social justice and public cooperation?

System justification theory (SJT) gives a reasonable explanation that people are motivated to defend and legitimize social systems even when doing so is not necessarily in their own interest (Jost et al., 2004; Jost and van der Toorn, 2012; van der Toorn and Jost, 2014). In particular, the more people feel dependent on an authority, the more they should be motivated to perceive him or her as legitimate (Kay et al., 2009). Outcome dependence is the extent to which someone is dependent on a powerful authority—the representative of a system—when that authority controls valued resources, which the social and/or material outcome the person desires (van der Toorn et al., 2011; Galinsky et al., 2015). Powerless people tend to justify rather than strive to change the hierarchical structures that disadvantage them (van der Toorn et al., 2015). Obviously, outcome dependence thus directly reflects the extent of system dependence, which triggers people to engage in system-justifying process (Kay and Friesen, 2011; Proudfoot and Kay, 2014).

Recent empirical evidence showed that outcome dependence was an independent contributor to the perceived legitimacy of authority apart from procedural fairness and outcome favorability (van der Toorn et al., 2011). In that research, perceived legitimacy was measured separately in terms of trust and confidence in authority, empowerment of authority, and deference to authority (see also Tyler, 2006). Regrettably, the logical connection between trust in authority and deference to authority was neglected. Two other experimental studies revealed that system dependence led participants to perceive policies as more desirable and reasonable, and to perceive the government as more responsible and benevolent (Kay et al., 2008, 2009). However, these studies only focused on individuals’ appraisals or attitudes toward legitimate authority instead of examining the indicators of behavior or behavioral intention when the subjects faced inequality or injustice (Gaucher et al., 2010).

Based on the foregoing arguments, we propose the following hypotheses:

H3: Outcome dependence moderates the relationship between social justice and public cooperation intention, namely:
H3a: Outcome dependence moderates the relationship between distributive justice and public cooperation intention. Specifically, under the condition of a high level of outcome dependence, the positive relationship between distributive justice and public cooperation intention will be weakened.
H3b: Outcome dependence moderates the relationship between procedural justice and public cooperation intention. Specifically, under the condition of a high level of outcome dependence, the positive relationship between procedural justice and public cooperation intention will be weakened.
H4: The moderation of outcome dependence functions through the mediating effect of competence-based trust, namely:
H4a: The interaction between distributive justice and outcome dependence is primarily related to competence-based trust, further influencing public cooperation intention. Specifically, under the condition of a high level of outcome dependence, the positive relationship between distributive justice and competence-based trust will be weakened.
H4b: The interaction between procedural justice and outcome dependence is primarily related to competence-based trust, further influences public cooperation intention. Specifically, under the condition of a high level of outcome dependence, the positive relationship between procedural justice and competence-based trust will be weakened.
H5: The moderation of outcome dependence functions through the mediating effect of motive-based trust, namely:
H5a: The interaction between distributive justice and outcome dependence is primarily related to motive-based trust, further influencing public cooperation intention. Specifically, under the condition of a high level of outcome dependence, the positive relationship between distributive justice and motive-based trust will be weakened.
H5b: The interaction between procedural justice and outcome dependence is primarily related to motive-based trust, further influencing public cooperation intention. Specifically, under the condition of a high level of outcome dependence, the positive relationship between procedural justice and motive-based trust will be weakened.

**FIGURE 1:**
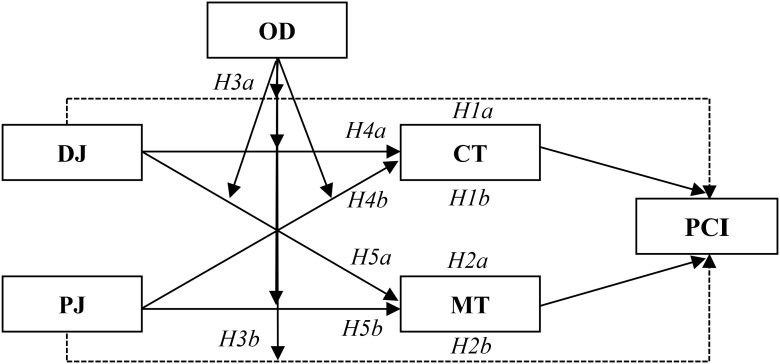
Research model and hypotheses. DJ, distributive justice; PJ, procedural justice; CT, competence-based trust; MT, motive-based trust; PCI, public cooperation intention; OD, outcome dependence.

In the present study, we aimed to elucidate the mechanism and the boundary for the effect of social justice on public cooperation intention. To achieve this goal, the complicated relationships among social justice, political trust, outcome dependence, and public cooperation intention were examined step by step. First, a dual-pathway model from social justice to public cooperation intention was built, and it connected through one pathway of competence-based trust and another pathway of motive-based trust (*H1* and *H2*). Second, the moderating role of outcome dependence in the dual-pathway model was explored (*H3, H4*, and *H5*). These five hypotheses, shown in **Figure 1**, were tested by an experiment (Study 1) and a survey (Study 2).^2^

## Study 1

The first study tested the mediating effect of political trust (i.e., *H1* and *H2*) and the moderating role of outcome dependence in the mediation model of public cooperation intention (i.e., *H3* to *H5*) in a laboratory environment.

For this purpose, we created an experimental scenario of public good dilemmas (PGDs). PGDs are the key multiple-person social dilemmas (Kollock, 1998), which are defined as “situations in which a non-cooperative course of action is (at times) tempting for each individual in that it yields superior (often short-term) outcomes for self, and if all pursue this non-cooperative course of action, all are (often in the longer-term) worse off than if all had cooperated” (van Lange et al., 2013). Further, the PGD is a give-some game of social dilemmas, in which each participant possesses some resources that are needed to provide an entity that all group members can use (van Lange et al., 2014). Compared with take-some games (i.e., resource dilemmas), PGDs are better able to reflect the degree of individual cooperation or prosocial behavior because the latter involves making more effort or giving up greater immediate benefits (van Dijke et al., 2018). In a public good experiment, each group member can keep resources or invest into a group account that represents the public good. Moreover, the individual contributes to the public account at the expense of his or her short-term gains (Fehr and Gintis, 2007).

### Method

#### Ethics Statement

All subjects gave a written informed consent in accordance with the Declaration of Helsinki, and their responses in the current study are all anonymous. In addition, this study was approved by the Institutional Review Board (IRB) of Institute of Psychology, Chinese Academy of Sciences.

#### Subjects and Design

A total of 320 Sun Yat-sen University junior undergraduates (45.9% male; mean age = 19.6, *SD* = 1.20) who were neither students of the Department of Psychology nor students of the School of Government participated in exchange for partial course credit in their social psychology classes.^3^ The subjects were randomly assigned to one of eight conditions in a 2 (distributive justice: high vs. low) × 2 (procedural justice: high vs. low) × 2 (outcome dependence: high vs. low) factorial design. There were 40 subjects under each condition.

#### Procedure and Materials

The experiment was conducted in laboratory rooms. Experimental materials were presented in the form of papers prepared in advance. All of the subjects were asked to read the scenarios separately and then fill out the questionnaires independently. The subjects did not interact with one another during the course of the experiment. The experimental procedure consisted of five steps:

First, a vignette of social justice was presented to the subjects. In particular, we used the classical paradigms of the ultimatum game (UG) and impunity game (IG) to manipulate social justice (Zhang, 2017). The topic of graduates’ employment was chosen to ensure the involvement of participants. The reading script first described a scenario:

A city government (Government A for short) decided to start a policy pilot of supporting the self-employment of graduates in its jurisdiction, district D (D for short). To this end, Government A allocated 100 million yuan of special funds to D. In order to fully mobilize the enthusiasm of the pilot area to carry out the policy, Government D is allowed to keep part of the funds for the necessary administrative expenses. However, according to the instruction of Government A, this 100 million must be allocated to every college graduate of D in the appropriate proportion. Otherwise, the funds from Government A will be reduced until withdrawn (if none of the graduates enjoy the preferential policy). There will be a total of 10,000 graduates in this year. Now suppose you are a graduating student of one university in D.

Next, distributive justice and procedural justice were manipulated in the pilot plan. The subjects learned about social justice information. Specifically:

After the discussion, Government D proposed the pilot plan as follows:

The proportion of allocation—90% of the total amount (i.e., 90 million yuan)—will be equally allocated to 10,000 graduates. That is, Government D will distribute 9,000 yuan per person at once, and this will be called “sponsorship for graduates’ self-employment.”

This material conveyed a high level of distributive justice to graduates. On the other hand, in the low level of distributive justice condition, the presented material was “*10% of the total amount (i.e., 10 million yuan) will be equally allocated to 10,000 graduates. That is, Government D will distribute 1,000 yuan per person at once.”* In sum, distributive justice was manipulated by setting a high (90%) or low (10%) ratio of money between graduates and Government D (Yamagishi et al., 2012).

In addition, procedural justice was manipulated by the paradigms of the UG and IG with materials about the rules of allocation. In the high procedural justice condition, the following material was used:

If you accept the offer from Government D, you will receive 9,000 (or 1,000) yuan. At the same time, Government D keeps 1,000 (or 9,000) yuan. If you reject the offer, you will receive 0 yuan, and Government D also keeps 0 yuan. In other words, your choice will decide the allocation of gains between you and Government D.

This rule comes from the UG, which is the most widely used decision paradigm for the study of fairness (Güth et al., 1982; Debove et al., 2016). In this experiment, Government D acted as the proposer, whereas a graduate acted as the responder. The proposer was in a strong and dominant position, but the responder had a voice. Voice effects are viewed as the most important factor in the judgment of procedural justice. A procedure in which the responder has a voice is more likely to be considered fair (Bobocel and Gosse, 2015; Vermunt and Steensma, 2016). Thus, giving individuals a voice is a conventional manipulation of procedural justice. Furthermore, both individuals’ allocation satisfaction and compliance with outcomes were found to be improved when they had a voice (Hibbing and Theiss-Morse, 2008). Moreover, in the UG, responders have the right of instrumental voice so that an individual has a significant impact on the outcome or decision (Lind and Tyler, 1988; Avery and Quiñones, 2002; Hibbing and Theiss-Morse, 2008). Because the responder can reject the proposal of the proposer, in order to punish him, they could indeed change the results. More importantly, a few past studies showed that the effect of instrumental voice led to higher procedural justice of the subjects than non-instrumental voice (Lind et al., 1990; Platow et al., 2013).

Compared to the instrumental voice of the responder in the UG, the responder may have a non-instrumental voice in the IG. The IG is seen as a variant of the UG, differing from the UG in two ways. First, the responder’s decision will only influence his own earnings. Second, whether the responder chooses to accept or reject the offer, the proposer can keep his money intact. In the face of an unfair offer by the proposer, if the responder refuses it, he will earn nothing (Bolton and Zwick, 1995; Yamagishi et al., 2009, 2012). Note that although the responder is given a voice in the IG, his rejection cannot affect the outcome of the distribution. This is the typical effect of non-instrumental voice (Lind et al., 1990; Platow et al., 2013). Hence, we used the IG paradigm to actualize the low level of procedural justice. Specifically, the following material was presented:

If you accept the offer by Government D, you will receive 9,000 (or 1,000) yuan. At the same time, Government D keeps 1,000 (or 9,000) yuan. If you reject the offer, you will receive 0 yuan. However, D government still keeps 1,000 (or 9,000) yuan. In other words, your choice only decides your own gains and does not influence the gains of Government D.

Third, outcome dependence was manipulated. In the condition of a high level of outcome dependence, the subjects read the following statement:

*Government A has already fully authorized to the pilot area. Government D can develop and implement the pilot plan independently.* In the condition of low level of outcome dependence, the subjects read the statement: *Government A is paying close attention to the pilot area. After the pilot plan is developed by Government D, Government A will evaluate it. If necessary, Government A can modify and improve this plan.*

The subjects had 15 min to read the experimental materials. Then, they were asked to fill out Questionnaire 1 (Q1), including the items for manipulation checks and the measurement of political trust.

Forth, after completing Q1, the subjects were exposed to a PGD scenario about self-employed graduates to examine public cooperation intention. The material of the PGD scenario was as follows:

In order to further encourage college graduates to start their own businesses, Government D intends to carry out a continuous funding scheme to support self-employed graduates. Specifically, Government D will set up a public fund named “Sunshine Business” (PFSB). Any self-employed graduate who qualifies will receive a certain amount of interest-free loans from the public fund in the first year. According to their actual situation, lenders are allowed to repay the principal in five years. PFSB raises funds in two ways—from the voluntary contributions of graduates and from the contributions of Government D. Government D will invest equivalent funds into this public account at the ratio of 1:1 with the voluntary contributions of graduates. For example, if 10,000 graduates donate 2,500 yuan per person (a total of 25 million yuan), Government D will also invest 25 million yuan, and therefore PFSB will have 50 million yuan in total. The money will be used solely to lend interest-free loans to support self-employed graduates. After the establishment of PFSB, all of the self-employed graduates can apply for interest-free loans. Of course, the number of beneficiaries depends on the financial resources of PFSB. In other words, when graduates contribute more, Government D provides more matching funds at the same time. Eventually, more self-employed graduates will benefit from PFSB. However, if no one contributes to the public accounts, there are also no equivalent funds from Government D. In short, whatever the contributions from graduates, Government D will invest the same amount. Once PFSB is established, it will benefit all of the self-employed graduates.

The subjects were asked to read the above material in 10 min, and then they decided whether or not to support Government D’s founding of PFSB in Questionnaire 2.

Finally, participants completed Questionnaire 3, which included measurements of control variables and personal information. Then they were thanked and debriefed.

#### Measures

##### Independent variables manipulation checks

To ensure that the distributive justice was effective, the subjects were asked to rate the following item on a 5-point scale (1 = *not at all*, 5 = *extremely*): “As a graduating student of one university in D, how fair do you think the outcome is based on the proportion of allocation from Government D?”

To ensure that the procedural justice was effective, the subjects were asked to rate the following item on a 5-point scale (1 = *not at all*, 5 = *extremely*): “As a graduating student of one university in D, how fair do you think the procedure is based on the rule of allocation from Government D?”

In addition, to ensure that the outcome dependence was successful, the subjects were asked to rate the following item on a 5-point scale (1 = *dependent very little*, 5 = *highly dependent*): “As a graduating student of one university in D, how dependent do you feel on Government D in the pilot plan?”

##### Political trust scale

Competence-based trust was assessed by three items on a 5-point scale, for example, “I think that Government D can make competent decisions about how to solve problems” (Tyler, 2011b). The response scale ranged from 1 (*disagree strongly*) to 5 (*agree strongly*). Cronbach’s α = 0.922.

Motive-based trust was assessed by three items on a 5-point scale—for instance, “I trust Government D to do what is best for college graduates like myself” (Tyler, 2011b). The response scale was “(1) *disagree strongly* to (5) *agree strongly.*” Cronbach’s α = 0.913.

##### Public cooperation intention

The subjects were asked to rate their extent of support for PFSB as a graduating student of a university in D (1 = *do not support at all*, 5 = *support completely*).

##### Control variables

Social value orientation (SVO) is defined as stable preferences that people assign to their own and others’ outcomes in situations of interdependence (van Lange et al., 1997; Balliet et al., 2009). SVO is not only a common personality trait that correlates with individual cooperation in social dilemmas (Balliet et al., 2009; van Lange et al., 2014), but also a justice-related personality trait (Gollwitzer and van Prooijen, 2016). Thus, SVO was used as the first control variable in this study. We measured SVO through the classical triple-dominance scales (van Lange et al., 1997).

Additionally, considering that the topic of the experimental scenario was college graduate enterprising, the subjects were asked whether they would start their own business after graduating. This variable was marked as the “self-employment intention.”

Lastly, gender was also viewed as a control variable.

### Results

#### Manipulation Checks

As expected, the manipulation checks for distributive justice, procedural justice, and outcome dependence were highly significant. First, the subjects in the high distributive justice condition reported that their outcome was more just than the subjects in the low distributive justice condition (*M*_s_ = 4.21 vs. 1.75, respectively), *F*(1, 318) = 1216.85, *p* < 0.001, η^2^ = 0.793. Second, the subjects in the high procedural justice condition reported that their procedure was more just than the subjects in the low procedural justice condition (*M*_s_ = 3.97 vs. 1.96, respectively), *F*(1, 318) = 717.98, *p* < 0.001, η^2^ = 0.693. Third, the subjects in the high outcome dependence condition reported that their outcome was more dependent than the subjects in the low outcome dependence condition (*M*_s_ = 4.28 vs. 1.81, respectively), *F*(1, 318) = 1451.83, *p* < 0.001, η^2^ = 0.820.

#### Descriptive Statistics

All of the subjects understood the scenario correctly and passed the context test successfully. Means, standard deviations, and intercorrelations are shown in **Table 1**. Regarding SVO statistics, there were 98 persons with “pro-self-orientation” (6 persons with “competitive orientation” and 92 persons with “individualistic orientation”) and 189 persons with “pro-social orientation” in this study (van Lange et al., 1997).

**Table 1:** Summary statistics and intercorrelations (Study 1).

Variable	*M*	*SD*	1	2	3	4	5	6	7	8
1. DJ manipulation	−	−								
2. PJ manipulation	−	−	< 0.01							
3. OD manipulation	−	−	< 0.01	< 0.01						
4. CT	3.16	1.00	0.63***	0.10^†^	0.36***					
5. MT	3.15	1.00	0.11*	0.69***	0.33***	0.49***				
6. PCI	3.27	1.16	0.25***	0.39***	0.49***	0.63***	0.73***			
7. SVO^a^	−	−	−0.04	−0.07	−0.02	0.01	−0.03	−0.002		
8. Self-employed^b^	−	−	< 0.01	0.15**	< 0.01	−0.03	0.09	−0.01	0.01	
9. Gender^c^	−	−	0.07	−0.08	0.04	0.05	−0.004	0.09	−0.001	0.02

*n = 320; ^∗∗∗^p < 0.001, ^∗∗^p < 0.01, ^∗^p < 0.05, ^†^p < 0.10. ^a^SVO is recorded as 1 = pro-self-orientation, 2 = pro-social orientation. ^b^Self-employment intention is recorded as 1 = yes, 2 = no. ^c^Gender is recorded as 1 = male, 2 = female.*

**Table 1** indicates that public cooperation intention was significantly related to social justice, political trust, and outcome dependence. In addition, no control variables were related to the dependent variable. Therefore, SVO, self-employment intention, and gender were not included in the following analysis.

#### Test of Mediation

**FIGURE 2:**
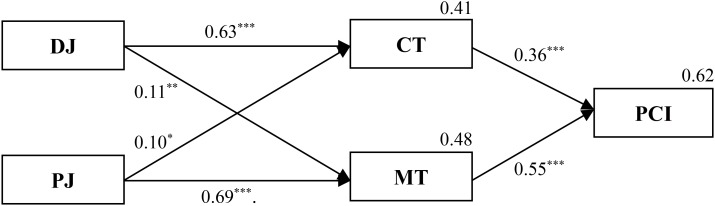
The dual-pathway model of public cooperation intention. *n* = 165; ^∗∗∗^*p* < 0.001, ^∗∗^*p* < 0.01, ^∗^*p* < 0.05. Model fit indices: χ^2^ = 3.01, *df* = 3, *p* = 0.39; CFI = 1.00, NFI = 1.00, RMSEA < 0.01.

We proposed a mediating effect of competence-based trust and motive-based trust on the relationship between distributive/procedural justice and public cooperation intention. A structural equation model (SEM) technique in AMOS 22.0 was used to test *H1* and *H2* (**Figure 1**). The SEM results revealed that a full mediation model adequately fit the data (χ^2^ = 3.01, *df* = 3, *p* = 0.39; CFI = 1.00, NFI = 1.00, and RMSEA < 0.01). The *R*^2^ of this model was 0.62. Standardized pathway coefficients among all of the variables are shown in **Figure 2**.

In addition, the standard errors and 90% confidence intervals for this mediation effect were generated by the bootstrapping option in AMOS 22.0. **Table 2** presents the indirect effects through competence-based trust and motive-based trust, confidence intervals, and decomposed indirect effect. The results showed that the indirect effects of both distributive justice and procedural justice on public cooperation intention were significant (β = 0.288, *p* = 0.015; and β = 0.414, *p* = 0.020; respectively), supporting our hypothesis that competence-based trust and motive-based trust would (fully) mediate the relationship between social justice and public cooperation intention. A further Sobel (1982) test indicated that both the indirect effect of distributive justice on public cooperation intention through competence-based trust (β = 0.226, *p* = 0.001) and the indirect effect of procedural justice on public cooperation intention through motive-based trust were significant (β = 0.377, *p* < 0.001). Consequently, Hypotheses 1 and 2 were supported.

**Table 2:** Indirect effects through competence-based trust and motive-based trust.

Relationship	Indirect effects (90% confidence interval; mediation by CT and MT)	Indirect effect through:	
		CT	MT
DJ→PCI	0.288^∗^ (0.213, 0.352)	0.226^∗∗^	0.062
PJ→PCI	0.414^∗^ (0.340, 0.477)	0.037	0.377^∗∗∗^

*^∗∗∗^p < 0.001, ^∗∗^p < 0.01, ^∗^p < 0.05.*

#### Test of Moderation

We tested the moderating role of outcome dependence in the relationship between social justice and public cooperation intention (i.e., *H3a* and *H3b*) via two-way ANOVA.

**FIGURE 3:**
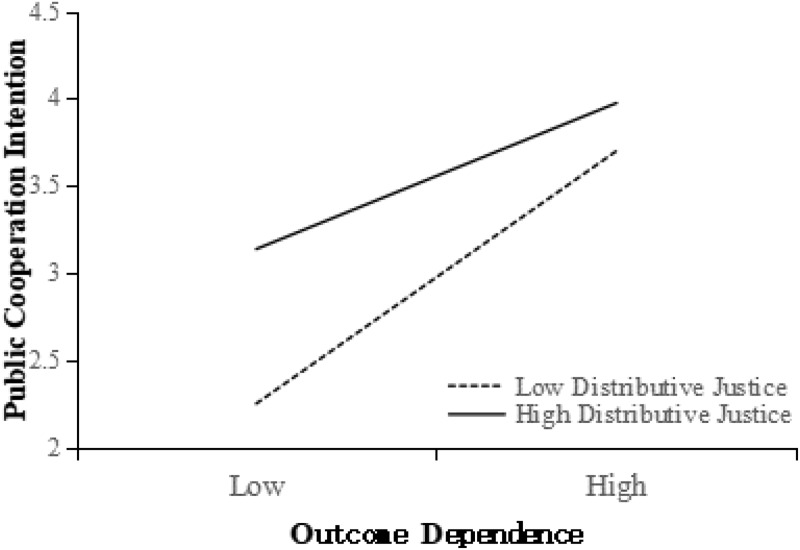
Interaction effect of distributive justice and outcome dependence on public cooperation intention.

First, an ANOVA of distributive justice, outcome dependence, and their interaction effect on public cooperation intention was performed (**Table 3**). The results indicated that the main effect of distributive justice and the main effect of outcome dependence were both significant (*p* < 0.001). More importantly, the distributive justice × outcome dependence interactions were also significant (*p* < 0.01). **Figure 3** shows the interaction effect of distributive justice and outcome dependence on public cooperation intention. More precisely, as predicted by *H3a*, the positive relationship between distributive justice and public cooperation intention was significantly stronger (comparing effect sizes) in the low outcome dependence condition (Wu and Wang, 2013)—*F*(1, 158) = 28.86, *p* < 0.001, η^2^= 0.154—than in the high outcome dependence condition, *F*(1, 158) = 4.03, *p* < 0.05, η^2^= 0.025.

**Table 3:** ANOVA of the effects of distributive justice, outcome dependence, and their interaction on public cooperation intention.

Variable	Sum of squares	*df*	Mean square	*F*
Distributive justice (A)	27.03	1	27.03	29.33^∗∗∗^
Outcome dependence (C)	104.65	1	104.65	113.55^∗∗∗^
A × C	7.50	1	7.50	8.14^∗∗^
Residual	291.24	316	0.92	
Total	3,843.00	320		

*Adjusted R^2^ = 0.317, F(3, 316) = 50.34, p < 0.001, η^2^ = 0.323. ^∗∗∗^p < 0.001, ^∗∗^p < 0.01.*

**FIGURE 4:**
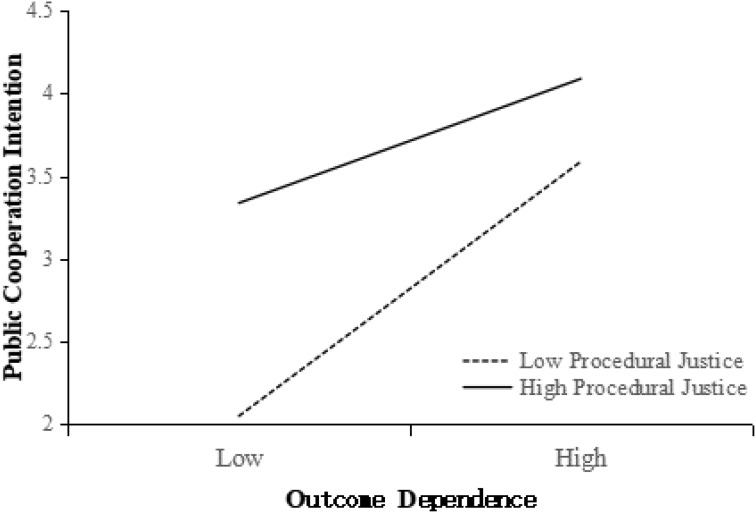
Interaction effect of procedural justice and outcome dependence on public cooperation intention.

Next, an ANOVA of procedural justice, outcome dependence, and their interaction effect on public cooperation intention was conducted (**Table 4**). The results revealed that the main effects of both procedural justice and outcome dependence were significant (*p* < 0.001). More importantly, the procedural justice × outcome dependence interactions were also significant (*p* < 0.001). **Figure 4** shows the interaction effect of procedural justice and outcome dependence on public cooperation intention. More specifically, as predicted by *H3b*, the positive relationship between procedural justice and public cooperation intention was significantly stronger in the low outcome dependence condition—*F*(1, 158) = 76.09, *p* < 0.001, η^2^= 0.325—than in the high outcome dependence condition, *F*(1, 158) = 14.14, *p* < 0.001, η^2^= 0.082.

**Table 4:** ANOVA testing the effects of procedural justice, outcome dependence, and their interaction on public cooperation intention.

Variable	Sum of squares	*df*	Mean square	*F*
Procedural justice (B)	63.90	1	63.90	80.95^∗∗∗^
Outcome dependence (C)	104.65	1	104.65	132.57^∗∗∗^
B × C	12.04	1	12.04	15.71^∗∗∗^
Residual	249.46	316	0.79	
Total	3,843.00	320		

*Adjusted R^2^= 0.415, F(3, 316) = 76.41, p < 0.001, η^2^= 0.420. ^∗∗∗^p < 0.001.*

In sum, outcome dependence moderated the effect of social justice on public cooperation intention, so that Hypothesis 3 was supported. Furthermore, to determine whether this moderation was mediated by political trust, a mediated moderation effect was examined. Specifically, we tested Hypotheses 4 and 5 by three-step regression analysis (Baron and Kenny, 1986; Muller et al., 2005; Ye and Wen, 2013).

First, we regressed public cooperation intention with distributive justice, outcome dependence, and distributive justice × outcome dependence. A significant coefficient (β = -0.23, *p* < 0.01) associated with distributive justice × outcome dependence implied that outcome dependence moderated the relationship between public cooperation intention and distributive justice (this result was consistent with that in **Table 3**).

**FIGURE 5:**
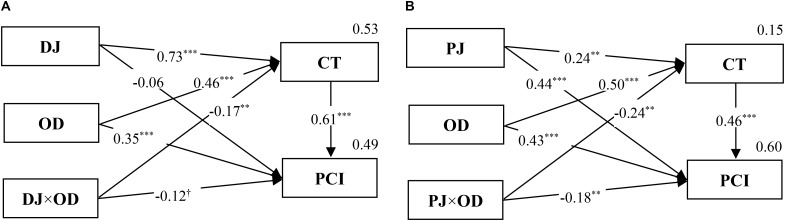
The mediated moderation model of DJ **(A)**, PJ **(B)**, OD, and CT on PCI (Study 1). *n* = 320; ^∗∗∗^*p* < 0.001, ^∗∗^*p* < 0.01, ^†^*p* < 0.10.

Second, we regressed competence-based trust with distributive justice, outcome dependence, and distributive justice × outcome dependence. Although the coefficients of both distributive justice (β = 0.73, *p* < 0.001) and outcome dependence (β = 0.46, *p* < 0.001) were significant, a significant coefficient (β = -0.17, *p* < 0.01) associated with distributive justice × outcome dependence suggested that outcome dependence moderated the relationship between competence-based trust and distributive justice (**Figure 5A**). Adjusted *R*^2^ = 0.53.

Third, regressed public cooperation intention on distributive justice, outcome dependence, competence-based trust and distributive justice × outcome dependence. Both coefficient (β = 0.61, *p* < 0.001) associated with competence-based trust and coefficient (β = -0.12, *p* < 0.10) associated with distributive justice × outcome dependence were significant, so the moderating effect is partially mediated (**Figure 5A**). Adjusted *R*^2^ = 0.49.

To explain the performance of the mediated moderation effect more clearly, we analyzed a simple effect of the relationship between distributive justice × outcome dependence interactions and competence-based trust. The results showed that the positive relationship between distributive justice and competence-based trust was significantly stronger in the low outcome dependence condition—*F*(1, 158) = 197.30, *p* < 0.001, η^2^= 0.555—than in the high outcome dependence condition, *F*(1, 158) = 88.22, *p* < 0.001, η^2^= 0.352. Hence, *H4a* was supported.

Using the same procedure and method, *H4b* was tested. The results indicated that the mediated moderation model was effective (**Figure 5B**). Additionally, the positive relationship between procedural justice and competence-based trust was significant in the low outcome dependence condition—*F*(1, 158) = 10.26, *p* < 0.01, η^2^= 0.061—but not in the high outcome dependence condition, *F*(1, 158) = 0.23, *ns*. Thus, *H4b* was supported.

**FIGURE 6:**
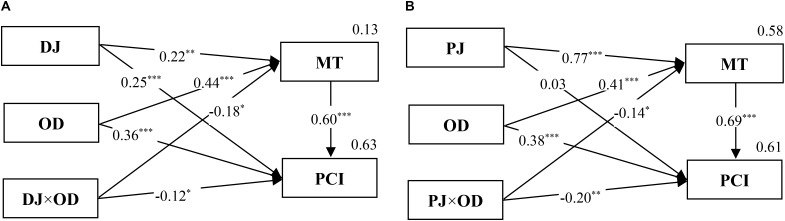
The mediated moderation model of DJ **(A)**, PJ **(B)**, OD, and MT on PCI (Study 1). *n* = 320; ^∗∗∗^*p* < 0.001, ^∗∗^*p* < 0.01, ^∗^*p* < 0.05.

Finally, Hypothesis 5 was tested in the same way. For *H5a*, the mediated moderation model of distributive justice, outcome dependence, and motive-based trust on public cooperation intention was supported (**Figure 6A**). The results revealed that the positive relationship between distributive justice and motive-based trust was significant in the low outcome dependence condition—*F*(1, 158) = 8.20, *p* < 0.01, η^2^= 0.049—but not in the high outcome dependence condition, *F*(1, 158) = 0.01, *ns*.

For *H5b*, the mediated moderation model of procedural justice, outcome dependence, and motive-based trust on public cooperation intention was again supported (**Figure 6B**). The results indicated that the positive relationship between procedural justice and motive-based trust was significantly stronger in the low outcome dependence condition—*F*(1, 158) = 248.40, *p* < 0.001, η^2^ = 0.611—than in the high outcome dependence condition, *F*(1, 158) = 127.80, *p* < 0.001, η^2^ = 0.447.

In conclusion, *H4* and *H5* were both supported. That is to say, the moderation of outcome dependence functioned through the mediating role of political trust.

### Discussion

We conducted two analyses in this study, including mediation and moderation. Mediation analysis confirmed the dual-pathway model of public cooperation intention. Specifically, a full mediation model was identified, indicating that the mediating effect of political trust on the relationship between social justice and public cooperation intention was strong when taken outcome dependence as a moderator. Moreover, the indirect effects of distributive and procedural justice on public cooperation intention, as shown by the SEM test, provided more evidence for full mediation relationships (Williams et al., 2009). Besides, as shown in **Figure 2**, the different justice types are not equally predictive of the two trust types. Specifically, distributive justice was more relevant to competence-based trust; while procedural justice was more relevant to motive-based trust. This result indirectly supported the classification of motivation for cooperation: both distributive justice and competence-based trust belong to instrumental motivations; whereas both procedural justice and motive-based trust belong to social motivations (Tyler, 2011b).

Moderation analysis showed that the interaction of outcome dependence and social justice (both distributive and procedural justice) not only influenced public cooperation intention, but were also related to political trust (both competence-based and motive-based trust). At the same time, the moderating mechanism of the outcome dependence effect on the relationship between social justice and public cooperation intention was very similar to the moderating mechanism of the outcome dependence effect on the relationship between social justice and political trust. Furthermore, the psychological effects of outcome dependence demonstrated that power affects how people feel, think, and act (Guinote, 2017).

In this experiment, we successfully manipulated social justice by UG and IG. Specifically, distributive justice of the responder (i.e., graduates) was derived from the fair distribution of cash by the proposer (i.e., government). This manipulation followed the UG paradigm, however, the distribution was not unequal between different groups of graduates but rather between the government itself and the graduate. In future studies, profit and distribution could be separated—so that the profit remains the same in both experimental condition (e.g., 1000), but the destitution differs (e.g., other graduates get the same amount of money versus a greater amount). On the other hand, procedural justice was manipulated by giving graduates an instrumental or non-instrumental voice to influence the government’s outcome. In particular, this power of the graduate was given by the government-an authority-proposed the pilot plan. This means that the difference in power between the two parties comes from their identity rather than experimental manipulation. So this manipulation is not so much the power of the graduate as it is his right. However, researchers have also used the UGs to instantiate power (Galinsky et al., 2015), since all subjects are anonymous individuals who do not know any other’s identity information, it easier to observe the proposer’s power over the responder, or vice versa. Therefore, though the experimental design was novel, future scholars needs to be more cautious in using UG and IG to manipulated social justice.

Up until this point, the research model we had proposed (**Figure 1**) was completely confirmed, and all of the hypotheses were supported by Study 1. A mediated moderation model from social justice to public cooperation intention was constructed by the experimental method. Next, we examine this mediated moderation effect in a real-world setting.

## Study 2

Study 2 was designed to examine the findings from Study 1 by questionnaire surveys in order to test the external or ecological validity of this research. Therefore, *H3* to *H7* were tested again by a field survey. We hoped that the results of the previous experiments could be replicated in a real-life situation.

In this study, we focused on the interaction between citizens and police. In China, the police are not only an example of legal authorities, but also an important department of the government. Moreover, people contact the police frequently in daily life when faced with all kinds of problems; as a Chinese saying goes, “call the police when you’re in trouble.” Therefore, we chose the police as the cooperative object for this study. There are two forms of voluntary efforts that actively aid the police (Tyler, 2011b). The first type consists of an individual providing relevant information to the police for dealing with a crime. The second type consists of an individual engaging in activities promoted by the police. Both forms of public cooperation intention were included in our survey.

### Method

#### Ethics Statement

All subjects gave a written informed consent in accordance with the Declaration of Helsinki, and their responses in the current study are all anonymous. In addition, this study was approved by the Institutional Review Board (IRB) of Institute of Psychology, Chinese Academy of Sciences.

#### Participants

A total of 1,310 adults who had lived in Guangzhou City, China for at least 6 months participated.^4^ All of the participants were interviewed over the telephone on the topic of “Survey on Police Service Satisfaction” concerning their perceptions of the police and crime in their neighborhood. The final sample included 1,240 participants (56% female; mean age = 39.8, *SD* = 16.76). This telephone survey program was conducted by the Public Opinion Research Center (PORC) of Sun Yat-sen University.

#### Measures

All of the variables (except outcome dependence) were assessed with five Likert-type scales and originated from Tyler (2011b). Specifically, the variables were as follows:

##### Distributive justice

Respondents were asked about the fairness of police service delivery to “people like you.” The response scale ranged from 1 (*extremely unfair*) to 5 (*extremely fair*).

##### Procedural justice

Respondents were asked to respond to the statement: “The police usually use fair procedures to decide how to handle the problems they deal with.” The response scale ranged from 1 (*disagree strongly*) to 5 (*agree strongly*).

##### Competence-based trust

Two items measured competence-based trust (Cronbach’s α = 0.713). One item was “You have confidence that the Guangzhou police can do their job well,” rated on a scale from 1 (*disagree strongly*) to 5 (*agree strongly*). Another item was: “How effective are the police in fighting crime in your neighborhood?” rated on a scale from 1 (*extremely ineffective*) to 5 (*extremely effective*).

##### Motive-based trust

Two items measured motive-based trust (Cronbach’s α = 0.754). Respondents were asked whether the police “Consider the views of people involved” and “Take into account the needs and concerns of the people they deal with.” The response scale ranged from 1 (*disagree strongly*) to 5 (*agree strongly*).

##### Outcome dependence

Outcome dependence in this case was assessed from a situation of relative powerlessness rather than one derived from a personal relationship with an authority figure, because the degree of dependence on the police depended on the degree of danger residents felt with respect to their neighborhood conditions (van der Toorn et al., 2011). The item was: “Overall, how high is the crime rate in your neighborhood?” The response scale ranged from 1 (*very low*) to 5 (*very high*).

##### Public cooperation intention

Public cooperation involved residents’ voluntary efforts to help the police (Cronbach’s α = 0.716). It was assessed by asking respondents, if the situation arose, how likely they would be to (1) call the police to report a crime; (2) help the police find someone suspected of a crime; (3) report dangerous or suspicious activity; (4) volunteer time to help the police; (5) patrol the streets as part of an organized group; or (6) volunteer to attend community meetings to discuss crime. The response scale ranged from 1 (*not at all likely*) to 5 (*very likely*).

Demographic variables can affect willingness to cooperate with the police, so the effects of respondents’ demographic information, such as age, gender, education, party identification, and length of residence in Guangzhou, were also examined.

### Results

As **Table 5** shows, there were significantly positive correlations among all of the research variables (v1–v6). Among the demographic variables, only age was significantly related to public cooperation intention.

**Table 5:** Summary of statistics and intercorrelations (Study 2).

Variable	*M*	*SD*	1	2	3	4	5	6	7	8	9	10
1. DJ	3.20	1.04										
2. PJ	3.36	1.05	0.63***									
3. OD	2.85	0.89	0.43***	0.45***								
4. CT	3.46	0.89	0.64***	0.62***	0.43***							
5. MT	3.31	0.95	0.61***	0.73***	0.60***	0.64***						
6. PCI	3.62	0.71	0.64***	0.62***	0.57***	0.64***	0.68***					
7. Age	39.82	16.76	0.06*	0.02	−0.01	0.13***	0.06	0.07*				
8. Gender^a^	−	−	−0.04	−0.01	−0.02	−0.03	−0.03	−0.05	< 0.01			
9. Education^b^	−	−	0.07*	0.05	< 0.01	0.01	0.01	0.02	−0.36***	0.03		
10. Party id^c^	−	−	0.09**	0.06*	0.01	0.08**	0.04	0.05	−0.13***	0.06*	0.27***	
11. Length	24.45	20.20	0.01	−0.03	−0.05	0.06*	−0.04	−0.02	0.73***	< 0.01	−0.27***	−0.18***

*n = 1,240; ^∗∗∗^p < 0.001, ^∗∗^p < 0.01, ^∗^p < 0.05. ^a^Gender is recorded as 1 = male, 2 = female. ^b^Education is partitioned into seven categories: illiterate (1), elementary school (2), junior high school (3), senior high school and technical secondary school (4), junior college (5), college (6), and postgraduate (7). ^c^Party identity is recorded as 1 = non-party, 2 = Democratic parties, 3 = CPC and League member.*

In order to confirm the moderating effect of outcome dependence on the relationship between social justice and public cooperation intention, we tested two liner regression models. Model 1 (*M1*) was a regression of public cooperation intention with distributive justice, outcome dependence, and their interaction. Model 2 (*M2*) was a regression of public cooperation intention with procedural justice, outcome dependence, and their interaction. **Table 6** shows the standard regression coefficients of all of the variables in *M1* and *M2*. The significant coefficient associated with distributive justice × outcome dependence (β = -0.20, *p* < 0.001) suggested that outcome dependence moderated the relationship between distributive justice and public cooperation intention. In addition, the significant coefficient associated with procedural justice × outcome dependence (β = -0.21, *p* < 0.001) suggested that outcome dependence also moderated the relationship between procedural justice and public cooperation intention. Hence, *H3* was supported.

**Table 6:** Linear regression of public cooperation intention with social justice, outcome dependence, and their interactions.

	*M1*	*M2*
Distributive justice (A)	0.42***	
Procedural justice (B)		0.38***
Outcome dependence (C)	0.36***	0.35***
A × C	−0.20***	
B × C		−0.21***
Age	0.12***	0.13***
Gender (RCV = female)	0.03	0.04*
**Education (RCV = illiterate)**		
*Elementary school*	0.13**	0.12**
*Junior high school*	0.18**	0.17**
*Senior high school*	0.24**	0.23**
*Junior college*	0.24**	0.23**
*College and above*	0.22**	0.21**
**Party id (RCV = non-party)**		
*Democratic parties*	−0.03	< 0.01
*CPC and League member*	−0.01	0.01
Length of residence	−0.08**	−0.08**
*N*	1,240	1,240
Adjusted *R^2^*	0.56	0.53

*n = 1,240; ^∗∗∗^p < 0.001, ^∗∗^p < 0.01, ^∗^p < 0.05.*

**FIGURE 7:**
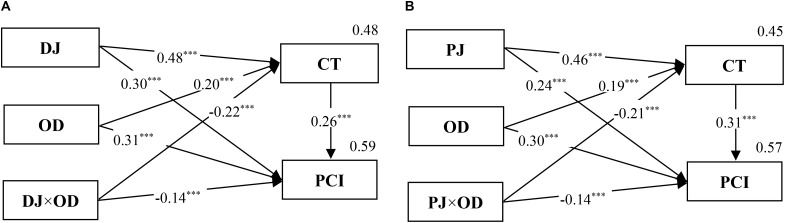
The mediated moderation model of DJ **(A)**, PJ **(B)**, OD, and CT on PCI (Study 2). *n* = 1,240; ^∗∗∗^*p* < 0.001.

**FIGURE 8:**
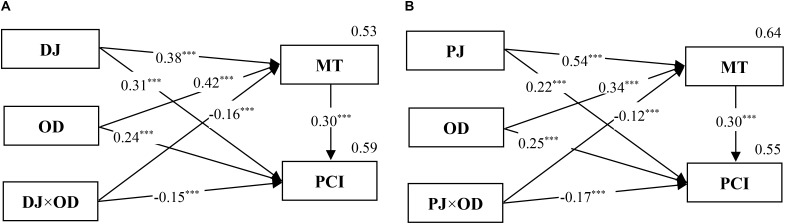
The mediated moderation model of DJ **(A)**, PJ **(B)**, OD, and MT on PCI (Study 2). *n* = 1,240; ^∗∗∗^*p* < 0.001.

A further analysis was conducted using three-step regression similar to that in Study 1. The results of the mediated moderation effects are shown in **Figures 7, 8**. In these two figures, all of the standard regression coefficients of relevant variables in Steps 2 and 3 are marked. Specifically, **Figure 7A** indicates that the moderating effect of outcome dependence on the relationship between distributive justice and public cooperation intention was mediated by competence-based trust. Moreover, **Figure 7B** shows that the moderating role of outcome dependence in the relationship between procedural justice and public cooperation intention was also mediated by competence-based trust. Thus, *H4* was supported. Similarly, **Figures 8A,B** indicate the moderating role of outcome dependence and the mediated moderation effect through motive-based trust. Thus, *H5* was supported.

### Discussion

Study 2 was designed to confirm the moderation effects found in Study 1 in a real-life scenario. Using cross-sectional data, Hypotheses 3, 4, and 5 were all supported repeatedly. In other words, the external validity of this research was confirmed, and the results were robust based on the experiment and survey.

In this study, the measures of social justice refer more to perceptions of justice rather than to actual justice, as in such context it may be more difficult to determine actual justice and one’s perception of justice have implications regardless of actual justice.

In addition, some demographic variables also affected public cooperation intention (**Table 6**). Overall, people with more education are more willing to cooperate with the police. This finding was consistent with another study indicating that “the more knowledge, the more rationality,” in which people with lower levels of education tended to complain but do nothing or turn to gathering their relatives to fight against the authority when suffering social injustice compared to people with relatively higher levels of education (Zhang et al., 2017). Interestingly, it was inconsistent that the correlation between length of residence and public cooperation intention was negative, while the correlation between age and public cooperation intention was positive. Older people tended to have a longer length of residence (the correlation between age and length of residence was 0.73, *p* < 0.001; **Table 5**). Future research should investigate and explain this phenomenon.

Last but not least, Study 2 can suffer from same-source bias or common method bias, as all data is from the same source (i.e., self-report), with the different variables correlated. Although this issue is ubiquitous in the survey, it is one limitation of this study. Future studies should use data from multiple sources (e.g., implicit association test, IAT) to increase the validity of the results.

## General Discussion and Conclusion

The question posed by *Science* implies that cooperation is vital to human evolution and society development. According to Sullivan et al. (2008), “The concept of cooperation is becoming more central to research in psychology and political sciences,” and understanding how public cooperation can be motivated is a core concern of social sciences (Tyler, 2011b). Therefore, in the present research, two studies explored the psychology of public cooperation, and new progress was made toward understanding this phenomenon.

Building upon the contemporary research on SET and fairness heuristic theory in the organizational context, this article proposed a new model to explain the mechanism of the relationship between social justice and public cooperation intention via political trust. We used a dual-pathway model from distributive and procedural justice to public cooperation intention through two pathways: competence-based trust and motive-based trust. The combination of these two paths, which is one of the two theoretical contributions of this paper, constituted a dual motivational system for individual participation in public cooperation. Regarding the mediation models, the present research confirmed the bridge of competence-based and motive-based political trust linking social justice and public cooperation intention was strong even when outcome dependence was considered as a moderator. Moreover, these results also indicated that distributive justice and procedural justice were both important for public cooperation intention; in fact, justice itself implies that normatively correct outcomes or resources have been assigned in a morally appropriate way (Cropanzano and Ambrose, 2015).

Drawing on SJT, we hypothesized that depending upon authorities who had the power to control one’s resources would strengthen public cooperation. A previous work demonstrated that outcome dependence was an independent factor to perceived legitimacy (van der Toorn et al., 2011). Our studies further found that outcome dependence moderated the relationship between social justice and public cooperation intention. Moreover, the moderation of outcome dependence functioned through the mediating effect of political trust. Studies 1 and 2 suggested that individuals were likely to trust the competence and motives of authorities, and consequently cooperate with them, when people depended on authorities. This work expends system justification research by revealing the boundary of system justification, especially in a collectivistic culture. This is another theoretical contribution of this paper. The existing research on outcome dependence has been conducted primarily in the Western context (particularly the United States) with individualist culture. However, collectivists are more deeply dependent on the system than individualists. Moreover, research has indicated that people from the East (e.g., China, Japan, Korea, and India) accept unequal distributions of power more easily than people in the West (e.g., United States, Canada, Australia, and Netherlands), and they are less likely to change the status quo (Pellegrini and Scandura, 2008). Individuals from high-power-distance and collectivistic societies tend to respect top management and follow their decisions rather than enjoy autonomous decision-making (Morrison and Milliken, 2000). Outcome dependence, therefore, is a general concept that exists in both West and East (Barkema et al., 2015).

Taken as a whole, our research integrated SET and SJT to establish a mediated moderation model from social justice to public cooperation intention via political trust and with the moderator of outcome dependence (**Figure 1**). In addition to this theoretical implication, the experiments featured a methodological innovation that provided two useful decision paradigms (i.e., the UG and IG) for manipulating social justice. Specifically, the UG was used to manipulate distributive justice, while the IG was used to manipulate procedural justice. To our knowledge, no other study has attempted this methodology, particularly the latter part (Zhang, 2017). These manipulation paradigms are easy to replicate, which can help avoid prejudices and operationalization complications that could threaten the validity of research in a public administration context.

Although the current studies yielded consistent results that contribute to the literature of justice, trust, and cooperation, they have some limitations, highlighting directions for future research. For example, although behavioral intention is the ideal substitute for actual behavior, it is not equal to actual behavior. Various factors limited our ability to examine actual behavior; for instance, the participants could not be asked to donate their real money in the public good dilemma scenario. Future studies could further examine the predictions of the dual-pathway model for public cooperation behavior. Moreover, future work may look for a more direct and powerful indicator of outcome dependence in daily life so that the effect of outcome dependence will be stronger in that condition.

The present findings have several practical implications. In order to promote public cooperation, authorities and/or governments should ensure the distributive justice and procedural justice of resource allocation—as Rawls (1971) wrote, “justice is the first virtue of social institutions.” Social justice can increase both competence-based trust and motive-based trust, which are ultimately conducive to public cooperation. Furthermore, it is beneficial to maintain the people’s dependence on the government. When authorities have the power to fulfill individuals’ desires, individuals are more willing to trust and cooperate with the authorities. Perhaps this is good news for governments. However, outcome dependence should originate from legitimate power, which “must be caged by the system” (Xi, 2014). Otherwise, public cooperation may turn into public resistance. If all of the authorities keep the above suggestions in mind and put them into practice, a harmonious society that is full of positive and effective interactions between people and the government will result.

## Author Contributions

All authors listed have made a substantial, direct and intellectual contribution to the work, and approved it for publication.

## Conflict of Interest Statement

The authors declare that the research was conducted in the absence of any commercial or financial relationships that could be construed as a potential conflict of interest.

## Footnotes

1 In this paper, “political trust” and “trust in government” are used interchangeably.

2 Although the relationship of the interaction between two different types of social justice and political trust on public cooperation may be an interesting topic, it is not the focus of our studies. However, considerating that the literature does find interactions between the two types of justice, or those of trust on dependent variables (such as McFarlin and Sweeney, 1992; Lu, 2014), we used experimental data from Study 1 to test the relationships of the interaction between justice types (i.e., distributive and procedural justice) and trust types (i.e., competence- and motive-based trust) on public cooperation intention. The results showed that the interactions of DJ^∗^PJ (*p* = 0.553) and CT^∗^MT (*p* = 0.338) were not significant.

3 Social psychology was an elective course for juniors (i.e., the third grade). Additionally, since junior students are about to graduate, they are more interested in employment issues than freshmen.

4 According to administrative districts’ proportion of the population in Guangzhou (data originated from the 2010 population census of China), the sample size of eight districts were: 99 people from Liwan District, 136 people from Yuexiu District, 178 people from Haizhu District, 172 people from Tianhe District, 104 people from Huangpu District, 259 people from Baiyun District, 172 people from Panyu District, and 120 people from Huadu District.

## References

[B1] Ajzen I. Fishbein M. (1980). *Understanding Attitudes and Predicting Social Behavior.* Englewood Cliffs, NJ: Prentice Hall.

[B2] Aryee S. Budhwar P. S. Chen Z. X. (2002). Trust as a mediator of the relationship between organizational justice and outcomes: test of a social exchange model. *J. Organ. Behav.* 23 267–285. 10.1002/job.138

[B3] Avery D. R. Quiñones M. A. (2002). Disentangling the effects of voice: the incremental roles of opportunity, behavior, and instrumentality in predicting procedural fairness. *J. Appl. Psychol.* 87 81–86. 10.1037/0021-9010.87.1.81 11916218

[B4] Balliet D. Parks C. Joireman J. (2009). Social value orientation and cooperation in social dilemmas: a meta-analysis. *Group Process. Intergroup Relat.* 12 533–547. 10.1177/1368430209105040

[B5] Barkema H. G. Chen X. P. George G. Luo Y. D. Tsui A. S. (2015). West meets east: new concepts and theories. *Acad. Manag. J.* 58 460–479. 10.5465/amj.2015.4021 27087864

[B6] Baron R. M. Kenny D. A. (1986). The moderator-mediator variable distinction in social psychological research: conceptual, strategic, and statistical considerations. *J. Pers. Soc. Psychol.* 51 1173–1182. 10.1037/0022-3514.51.6.1173 3806354

[B7] Bobocel D. R. Gosse L. (2015). “Procedural justice: a historical review and critical analysis,” in *The Oxford Handbook of Justice in the Workplace*, eds Cropanzano R. S. Ambrose M. L. (Oxford: Oxford University Press), 51–87.

[B8] Bolton G. E. Zwick R. (1995). Anonymity versus punishment in ultimatum bargaining. *Games Econ. Behav.* 10 95–121. 10.1006/game.1995.1026

[B9] Castillo J. C. Palacios D. Joignant A. Tham M. (2015). Inequality, distributive justice and political participation: an analysis of the case of Chile. *Bull. Lat. Am. Res.* 34 486–502. 10.1111/blar.12369

[B10] Chanley V. A. Rudolph T. J. Rahn W. M. (2000). The origins and consequences of public trust in government: a time series analysis. *Public Opin. Q.* 64 239–256. 11114267 10.1086/317987

[B11] Colquitt J. A. Conlon D. E. Wesson M. J. Porter C. O. Ng K. Y. (2001). Justice at the millennium: a meta-analytic review of 25 years of organizational justice research. *J. Appl. Psychol.* 86 425–445. 10.1037/0021-9010.86.3.425 11419803

[B12] Colquitt J. A. LePine J. A. Piccolo R. F. Zapata C. P. Rich B. L. (2012). Explaining the justice-performance relationship: trust as exchange deepener or trust as uncertainty reducer? *J. Appl. Psychol.* 97 1–15. 10.1037/a0025208 21910516

[B13] Colquitt J. A. Long D. M. Rodell J. B. Halvorsen-Ganepola M. D. K. (2015). Adding the “in” to justice: a qualitative and quantitative investigation of the differential effects of justice rule adherence and violation. *J. Appl. Psychol.* 100 278–297. 10.1037/a0038131 25420056

[B14] Colquitt J. A. Rodell J. B. (2015). “Measuring justice and fairness,” in *The Oxford Handbook of Justice in the Workplace*, eds Cropanzano R. S. Ambrose M. L. (Oxford: Oxford University Press), 187–202.

[B15] Colquitt J. A. Scott B. A. Rodell J. B. Long D. M. Zapata C. P. Conlon D. E. (2013). Justice at the millennium, a decade later: a meta-analytic test of social exchange and affect-based perspectives. *J. Appl. Psychol.* 98 199–236. 10.1037/a0031757 23458336

[B16] Colquitt J. A. Zipay K. P. (2015). Justice, fairness, and employee reactions. *Annu. Rev. Organ. Psychol. Organ. Behav.* 2 75–99. 10.1146/annurev-orgpsych-032414-111457

[B17] Cropanzano R. Anthony E. L. Daniels S. R. Hall A. V. (2017). “Entity justice and entity injustice: a review and conceptual extension,” in *Organizational Justice: International Perspectives and Conceptual Advances*, eds Moliner C. Cropanzano R. Martínez-Tur V. (London: Routledge), 207–243.

[B18] Cropanzano R. Mitchell M. S. (2005). Social exchange theory: an interdisciplinary review. *J. Manag.* 31 874–900. 10.1111/ddg.13605 29998596

[B19] Cropanzano R. S. Ambrose M. L. (2015). “Organizational justice: where we have been and where we are going,” in *The Oxford Handbook of Justice in the Workplace*, eds Cropanzano R. S. Ambrose M. L. (Oxford: Oxford University Press), 3–13.

[B20] Debove S. Baumard N. André J. B. (2016). Models of the evolution of fairness in the ultimatum game: a review and classification. *Evol. Hum. Behav.* 37 245–254. 10.1016/j.evolhumbehav.2016.01.001

[B21] Fehr E. Gintis H. (2007). Human motivation and social cooperation: experimental and analytical foundations. *Annu. Rev. Sociol.* 33 43–64. 10.1146/annurev.soc.33.040406.131812

[B22] Feldman D. L. (2015). The legitimacy of U. S. government agency power. *Public Adm. Rev.* 75 75–84. 10.1111/puar.12279

[B23] Galinsky A. D. Rucker D. D. Magee J. C. (2015). “Power: past findings, present considerations, and future directions,” in *APA Handbook of Personality and Social Psychology: Vol. 3. Interpersonal Relations*, eds Mikulincer M. Shaver P. R. (Washington, DC: American Psychological Association), 421–460 10.1037/14344-016

[B24] Gaucher D. Kay A. C. Laurin K. (2010). “The power of the status quo,” in *The Psychology of Justice and Legitimacy: The Ontario Symposium*, eds Bobocel D. R. Kay A. C. Zanna M. P. Olson J. M. (New York, NY: Psychology Press), 151–172.

[B25] Gollwitzer M. van Prooijen J.-W. (2016). “Psychology of justice,” in *Handbook of Social Justice Theory and Research*, eds Sabbagh C. Schmitt M. (New York, NY: Springer), 61–82. 10.1007/978-1-4939-3216-0_4

[B26] Guinote A. (2017). How power affects people: activating, wanting, and goal seeking. *Annu. Rev. Psychol.* 68 353–381. 10.1146/annurev-psych-010416-044153 27687123

[B27] Güth W. Schmittberger R. Schwarze B. (1982). An experimental analysis of ultimatum bargaining. *J. Econ. Behav. Organ.* 3 367–388. 10.1016/0167-2681(82)90011-7

[B28] Hetherington M. J. (1999). The effect of political trust on the presidential vote, 1968-96. *Am. Polit. Sci. Rev.* 93 311–326. 10.2307/2585398

[B29] Hetherington M. J. (2005). *Why Trust Matters: Declining Political Trust and the Demise of American Liberalism.* Princeton, NJ: Princeton University Press.

[B30] Hibbing J. R. Theiss-Morse E. (2008). “Voice, validation and legitimacy,” in *Cooperation: The Political Psychology of Effective Human Interaction*, eds Sullivan B. A. Snyder M. Sullivan J. L. (Malden, MA: Blackwell Publishing, Ltd.), 123–142.

[B31] Huq A. Z. Tyler T. R. Schulhofer S. J. (2011). Why does the public cooperate with law enforcement? The influence of the purposes and targets of policing? *Psychol. Public Policy Law* 17 419–450. 10.1037/a0023367

[B32] Jasso G. Törnblom K. Y. Sabbagh C. (2016). “Distributive justice,” in *Handbook of Social Justice Theory and Research*, eds Sabbagh C. Schmitt M. (New York, NY: Springer), 201–218. 10.1007/978-1-4939-3216-0_11

[B33] Jost J. T. Banaji M. R. Nosek B. A. (2004). A decade of system justification theory: accumulated evidence of conscious and unconscious bolstering of the status quo. *Polit. Psychol.* 25 881–919. 10.1111/j.1467-9221.2004.00402.x

[B34] Jost J. T. van der Toorn J. (2012). “System justification theory,” in *Handbook of Theories of Social Psychology*, eds van Lange P. A. M. Kruglanski A. W. Higgins E. T. (London: Sage), 313–343. 10.4135/9781446249222.n42

[B35] Kay A. C. Friesen J. (2011). On social stability and social change: understanding when system justification does and does not occur. *Curr. Dir. Psychol. Sci.* 20 360–364. 10.1177/0963721411422059

[B36] Kay A. C. Gaucher D. Napier J. L. Callan M. J. Laurin K. (2008). God and the government: testing a compensatory control mechanism for the support of external systems. *J. Pers. Soc. Psychol.* 95 18–35. 10.1037/0022-3514.95.1.18 18605849

[B37] Kay A. C. Gaucher D. Peach J. M. Laurin K. Friesen J. Zanna M. P. (2009). Inequality, discrimination, and the power of the status quo: Direct evidence for a motivation to see the way things are as the way they should be. *J. Pers. Soc. Psychol.* 97 421–434. 10.1037/a0015997 19685999

[B38] Kennedy D. Norman C. (2005). What don’t we know? *Science* 309:75. 10.1126/science.309.5731.75 15994521

[B39] Kim S. E. (2005). The role of trust in the modern administrative state: an integrative model. *Adm. Soc.* 37 611–635. 10.1177/0095399705278596

[B40] Kollock P. (1998). Social dilemmas: the anatomy of cooperation. *Annu. Rev. Sociol.* 24 183–214. 10.1146/annurev.soc.24.1.183

[B41] Konovsky M. A. Pugh S. D. (1994). Citizenship behavior and social exchange. *Acad. Manag. J.* 37 656–669. 10134637

[B42] Levi M. (1998). “A state of trust,” in *Trust and Governance*, eds Braithwaite A. Levi M. (New York, NY: Sage), 77–101.

[B43] Levi M. Stoker L. (2000). Political trust and trustworthiness. *Annu. Rev. Polit. Sci.* 3 475–507. 10.1146/annurev.polisci.3.1.475

[B44] Li L. J. (2004). Political trust in rural China. *Mod. China* 30 228–258. 10.1177/0097700403261824

[B45] Li L. J. (2008). Political trust and petitioning in the Chinese countryside. *Comp. Polit.* 40 209–226. 10.5129/001041508X12911362382832

[B46] Lind E. A. (2001). “Fairness heuristic theory: justice judgments as pivotal cognitions in organizational relations,” in *Advances in Organizational Justice*, eds Greenberg J. Cropanzano R. (Stanford, CA: Stanford University Press), 56–88.

[B47] Lind E. A. Kanfer R. Earley P. C. (1990). Voice, control, and procedural justice: instrumental and noninstrumental concerns in fairness judgments. *J. Pers. Soc. Psychol.* 59 952–959. 10.1037/0022-3514.59.5.952

[B48] Lind E. A. Tyler T. R. (1988). *The Social Psychology of Procedural Justice.* Berlin: Springer 10.1007/978-1-4899-2115-4

[B49] Lu J. (2014). A cognitive anatomy of political trust and respective bases: evidence from a two-city survey in China. *Polit. Psychol.* 35 477–494. 10.1111/pops.12058

[B50] Marien S. Hooghe M. (2011). Does political trust matter? An empirical investigation into the relation between political trust and support for law compliance. *Eur. J. Polit. Res.* 50 267–291. 10.1111/j.1475-6765.2010.01930.x

[B51] Martin J. (1986). “The tolerance of injustice,” in *Relative Deprivation and Social Comparison: The Ontario Symposium* Vol. 4 eds Olson J. M. Herman C. P. Zanna M. P. (Hillsdale, NJ: Lawrence Erlbaum), 217–242.

[B52] McFarlin D. B. Sweeney P. D. (1992). Distributive and procedural justice as predictors of satisfaction with personal and organizational outcomes. *Acad. Manag. J.* 35 626–637.

[B53] Morrison E. W. Milliken F. J. (2000). Organizational silence: a barrier to change and development in a pluralistic world. *Acad. Manag. Rev.* 25 706–731. 10.5465/amr.2000.3707697

[B54] Muller D. Judd C. M. Yzerbyt V. Y. (2005). When moderation is mediated and mediation is moderated. *J. Pers. Soc. Psychol.* 89 852–863. 10.1037/0022-3514.89.6.852 16393020

[B55] Murphy K. (2014). “Procedure justice, legitimacy, and policing,” in *Encyclopedia of Criminology and Criminal Justice*, eds Bruinsma G. Weisburd D. (New York, NY: Springer), 4024–4034. 10.1007/978-1-4614-5690-2_65

[B56] Nagin D. S. Telep C. W. (2017). Procedural justice and legal compliance. *Annu. Rev. Law Soc. Sci.* 13 1 1–1.24. 10.1146/annurev-lawsocsci-110316-113310

[B57] Pellegrini E. K. Scandura T. A. (2008). Paternalistic leadership: a review and agenda for future research. *J. Manag.* 34 566–593. 10.1177/0149206308316063

[B58] Platow M. J. Eggins R. A. Chattopadhyay R. Brewer G. Hardwick L. Milsom L. (2013). Two experimental tests of relational models of procedural justice: non-instrumental voice and authority group membership. *Br. J. Soc. Psychol.* 52 361–376. 10.1111/j.2044-8309.2011.02083.x 22251431

[B59] Proudfoot D. Kay A. C. (2014). System justification in organizational contexts: how a Motivated preference for the status quo can affect organizational attitudes and behaviors. *Res. Organ. Behav.* 34 173–187. 10.1016/j.riob.2014.03.001

[B60] Proudfoot D. Lind E. A. (2015). “Fairness heuristic theory, the uncertainty management model, and fairness at work,” in *The Oxford Handbook of Justice in the Workplace*, eds Cropanzano R. S. Ambrose M. L. (Oxford: Oxford University Press), 371–385.

[B61] Rankin L. E. Tyler T. R. (2009). Justice and cooperation. *Neth. J. Psychol.* 65 146–154. 10.1007/BF03080137

[B62] Rawls J. (1971). *A Theory of Justice.* Oxford: Oxford University Press.

[B63] Rothmund T. Becker J. C. Jost J. T. (2016). “The psychology of social justice in political thought and action,” in *Handbook of Social Justice Theory and Research*, eds Sabbagh C. Schmitt M. (New York, NY: Springer), 275–291.

[B64] Rudolph T. J. Evans J. (2005). Political trust, ideology, and public support for government spending. *Am. J. Polit. Sci.* 49 660–671. 10.1111/j.1540-5907.2005.00148.x

[B65] Scholz J. T. Lubell M. (1998). Trust and taxpaying: testing the heuristic approach to collective action. *Am. J. Polit. Sci.* 42 398–417. 10.2307/2991764

[B66] Sobel M. E. (1982). “Asymptotic confidence intervals for indirect effects in structural equation models,” in *Sociological Methodology* Vol. 13 ed. Leinhardt S. (San Francisco: John Wiley & Sons), 290–312.

[B67] Sullivan B. A. Snyder M. Sullivan J. L. (2008). *Cooperation: The Political Psychology of Effective Human Interaction.* Malden, MA: Blackwell Publishing, Ltd.

[B68] Sunshine J. Tyler T. R. (2003). The role of procedural justice and legitimacy in shaping public support for policing. *Law Soc. Rev.* 37 513–548. 10.1177/1529100615617791 26635334

[B69] Sweeney P. D. McFarlin D. B. (1993). Workers’ evaluations of the “ends” and the “means”: an examination of four models of distributive and procedural justice. *Organ. Behav. Hum. Decis. Process.* 55 23–40. 10.1006/obhd.1993.1022

[B70] Törnblom K. Y. Kazemi A. (2015). “Distributive justice: revisiting past statements and reflecting on future prospects,” in *The Oxford Handbook of Justice in the Workplace*, eds Cropanzano R. S. Ambrose M. L. (Oxford: Oxford University Press), 15–50.

[B71] Tyler T. R. (2000). Social justice: outcome and procedure. *Int. J. Psychol.* 35 117–125. 10.1080/002075900399411

[B72] Tyler T. R. (2006). *Why People Obey the Law: Procedural Justice, Legitimacy, and Compliance*, 2nd Edn. Princeton, NJ: Princeton University Press.

[B73] Tyler T. R. (2010). “Legitimacy and rule adherence: a psychological perspective on the antecedents and consequences of legitimacy,” in *The Psychology of Justice and Legitimacy: The Ontario Symposium*, eds Bobocel D. R. Kay A. C. Zanna M. P. Olson J. M. (New York, NY: Psychology Press), 251–272.

[B74] Tyler T. R. (2011a). Trust and legitimacy: policing in the USA and Europe. *Eur. J. Criminol.* 8 254–266. 10.1177/1477370811411462

[B75] Tyler T. R. (2011b). *Why People Cooperate.* Princeton, NJ: Princeton University Press.

[B76] Tyler T. R. (2012). Justice and effective cooperation. *Soc. Justice Res.* 25 355–375. 10.1007/s11211-012-0168-5

[B77] Tyler T. R. (2017). Procedural justice and policing: a rush to judgment? *Annu. Rev. Law Soc. Sci.* 13 2 1–2.25. 10.1146/annurev-lawsocsci-110316-113318

[B78] Tyler T. R. Jackson J. Bradford B. (2014). “Procedure justice and cooperation,” in *Encyclopedia of Criminology and Criminal Justice*, eds Bruinsma G. Weisburd D. (New York: Springer), 4011–4024. 10.1007/978-1-4614-5690-2_64

[B79] van Damme A. Pauwels L. Svensson R. (2015). Why do Swedes cooperate with the police? A SEM analysis of Tyler’s procedural justice model. *Eur. J. Crim. Policy Res.* 21 15–33. 10.1007/s10610-013-9224-4

[B80] van der Toorn J. M. Feinberg M. Jost J. T. Kay A. C. Tyler T. R. Willer R. (2015). A sense of powerlessness fosters system justification: implications for the legitimation of authority, hierarchy, and government. *Polit. Psychol.* 36 93–110. 10.1111/pops.12183

[B81] van der Toorn J. M. Jost J. T. (2014). Twenty years of system justification theory: introduction to the special issue on “Ideology and system justification processes”. *Group Process. Intergroup Relat.* 17 413–419. 10.1177/1368430214531509

[B82] van der Toorn J. M. Tyler T. R. Jost J. (2011). More than fair: outcome dependence, system justification, and the perceived legitimacy of authority figures. *J. Exp. Soc. Psychol.* 47 127–138. 10.1016/j.jesp.2010.09.003

[B83] van Dijke M. Cremer D. D. Langendijk G. Anderson C. (2018). Ranking low, feeling high: how hierarchical position and experienced power promote prosocial behavior in response to procedural justice. *J. Appl. Psychol.* 103 164–181. 10.1037/apl0000260 28933910

[B84] van Lange P. A. M. Balliet D. Parks C. D. van Vugt M. (2014). *Social Dilemmas: The Psychology of Human Cooperation.* Oxford: Oxford University Press.

[B85] van Lange P. A. M. De Bruin E. M. N. Otten W. Joireman J. A. (1997). Development of prosocial, individualistic, and competitive orientations: theory and preliminary evidence. *J. Pers. Soc. Psychol.* 73 733–746. 10.1037/0022-3514.73.4.733 9325591

[B86] van Lange P. A. M. Joireman J. Parks C. D. van Dijk E. (2013). The psychology of social dilemmas: a review. *Organ. Behav. Hum. Decis. Process.* 120 125–141. 10.1016/j.obhdp.2012.11.003

[B87] VanVugt M. Snyder M. Tyler T. R. Biel A. (2000). *Cooperation in Modern Society: Promoting the Welfare of Communities, States and Organizations.* New York, NY: Routledge.

[B88] Verboon P. Goslinga S. (2009). The role of fairness in tax compliance. *Neth. J. Psychol.* 65 136–145. 10.1007/BF03080136

[B89] Vermunt R. Steensma H. (2016). “Procedural justice,” in *Handbook of Social Justice Theory and Research*, eds Sabbagh C. Schmitt M. (New York, NY: Springer), 219–236. 10.1007/978-1-4939-3216-0_12

[B90] Williams L. J. Vandenberg R. J. Edwards J. R. (2009). Structural equation modeling in management research: a guide for improved analysis. *Acad. Manag. Ann.* 3 543–604. 10.5465/19416520903065683

[B91] Wright S. C. (2001). “Restricted intergroup boundaries: tokenism, ambiguity, and the tolerance of injustice,” in *The Psychology of Legitimacy: Emerging Perspectives on Ideology, Justice, and Intergroup Relations*, eds Jost J. T. Major B. (New York, NY: Cambridge University Press), 223–254.

[B92] Wu X. N. Wang E. P. (2013). Outcome favorability as a boundary condition to voice effect on people’s reactions to public policymaking. *J. Appl. Soc. Psychol.* 43 329–337. 10.1111/j.1559-1816.2012.01002.x

[B93] Xi J. P. (2014). *Xi Jingping: The Governance of China.* Beijing: Foreign Languages Press.

[B94] Yamagishi T. Horita Y. Mifune N. Hashimoto H. Li Y. Shinada M. (2012). Rejection of unfair offers in the ultimatum game is no evidence of strong reciprocity. *Proc. Natl. Acad. Sci. U.S.A.* 109 20364–20368. 10.1073/pnas.1212126109 23188801 PMC3528519

[B95] Yamagishi T. Horita Y. Takagishi H. Shinada M. Tanida S. Cook K. S. (2009). The private rejection of unfair offers and emotional commitment. *Proc. Natl. Acad. Sci. U.S.A.* 106 11520–11523. 10.1073/pnas.0900636106 19564602 PMC2703666

[B96] Ye B. J. Wen Z. L. (2013). A discussion on testing methods for mediated moderation models: discrimination and integration (in Chinese). *Acta Psychol. Sin.* 45 1050–1060. 10.3724/SP.J.1041.2013.01050

[B97] Zhang S. W. (2017). Social justice, institutional trust and public cooperation intention (in Chinese). *Acta Psychol. Sin.* 49 794–813. 10.3724/SP.J.1041.2017.00794

[B98] Zhang S. W. Lin J. Y. Zhou J. (2017). The more knowledge, the more rationality: relationship between level of education and individual coping behavior intention in social injustice context (in Chinese). *J. Emerg. Manag.* 1 110–119.

[B99] Zhang S. W. Wang E. P. Zhou J. (2010). Relative deprivation and relative gratification: the motivation of Chinese mass incidents (in Chinese). *J. Public Manag.* 7 95–102.

[B100] Zhou J. Wang E. P. (2012). Pathways to hostile collective action: the roles of general attitudes toward the advantaged group and situational anger. *Psych J.* 1 90–100. 10.1002/pchj.3 26272760

